# Proteomic profiling of breast cancer metabolism identifies SHMT2 and ASCT2 as prognostic factors

**DOI:** 10.1186/s13058-017-0905-7

**Published:** 2017-10-11

**Authors:** Stephan Bernhardt, Michaela Bayerlová, Martina Vetter, Astrid Wachter, Devina Mitra, Volker Hanf, Tilmann Lantzsch, Christoph Uleer, Susanne Peschel, Jutta John, Jörg Buchmann, Edith Weigert, Karl-Friedrich Bürrig, Christoph Thomssen, Ulrike Korf, Tim Beissbarth, Stefan Wiemann, Eva Johanna Kantelhardt

**Affiliations:** 1 Division of Molecular Genome Analysis, German Cancer Research Center (DKFZ), Im Neuenheimer Feld 580, 69120 Heidelberg, Germany; 2 Department of Medical Statistics, University Medical Center Goettingen, Humboldtallee 32, 37073 Goettingen, Germany; 3 Department of Gynaecology, Martin-Luther-University, Halle-Wittenberg, Ernst-Grube-Str. 40, 06120 Halle (Saale), Germany; 4 Department of Gynaecology, Hospital Fuerth, Jakob-Henle-Str. 1, 90768 Fuerth, Germany; 5 Department of Gynaecology, Hospital St. Elisabeth and St. Barbara, Mauerstr. 5, 06110 Halle (Saale), Germany; 6 Onkologische Praxis Uleer, Bahnhofstr. 5, 31134 Hildesheim, Germany; 7 Department of Gynaecology, St. Bernward Hospital, Treibestr. 9, 31134 Hildesheim, Germany; 8 Department of Gynaecology, Helios Hospital Hildesheim, Weinberg 1, 31134 Hildesheim, Germany; 9 Institute of Pathology, Hospital Martha-Maria, Roentgenstraße 1, 06120 Halle (Saale), Germany; 10 Institute of Pathology, Hospital Fuerth, Jakob-Henle-Str. 1, 90768 Fuerth, Germany; 11 Institute of Pathology Hildesheim, Senator-Braun-Allee 35, 31135 Hildesheim, Germany; 12 Institute of Medical Epidemiology, Biostatistics and Informatics, Martin-Luther-University, Halle-Wittenberg, Magdeburgerstr. 8, 06120 Halle (Saale), Germany

**Keywords:** Protein arrays, Breast cancer, Cancer metabolism, SHMT2, SLC1A5

## Abstract

**Background:**

Breast cancer tumors are known to be highly heterogeneous and differences in their metabolic phenotypes, especially at protein level, are less well-understood. Profiling of metabolism-related proteins harbors the potential to establish new patient stratification regimes and biomarkers promoting individualized therapy. In our study, we aimed to examine the relationship between metabolism-associated protein expression profiles and clinicopathological characteristics in a large cohort of breast cancer patients.

**Methods:**

Breast cancer specimens from 801 consecutive patients, diagnosed between 2009 and 2011, were investigated using reverse phase protein arrays (RPPA). Patients were treated in accordance with national guidelines in five certified German breast centers. To obtain quantitative expression data, 37 antibodies detecting proteins relevant to cancer metabolism, were applied. Hierarchical cluster analysis and individual target characterization were performed. Clustering results and individual protein expression patterns were associated with clinical data. The Kaplan-Meier method was used to estimate survival functions. Univariate and multivariate Cox regression models were applied to assess the impact of protein expression and other clinicopathological features on survival.

**Results:**

We identified three metabolic clusters of breast cancer, which do not reflect the receptor-defined subtypes, but are significantly correlated with overall survival (OS, *p* ≤ 0.03) and recurrence-free survival (RFS, *p* ≤ 0.01). Furthermore, univariate and multivariate analysis of individual protein expression profiles demonstrated the central role of serine hydroxymethyltransferase 2 (SHMT2) and amino acid transporter ASCT2 (SLC1A5) as independent prognostic factors in breast cancer patients. High SHMT2 protein expression was significantly correlated with poor OS (hazard ratio (HR) = 1.53, 95% confidence interval (CI) = 1.10–2.12, *p* ≤ 0.01) and RFS (HR = 1.54, 95% CI = 1.16–2.04, *p* ≤ 0.01). High protein expression of ASCT2 was significantly correlated with poor RFS (HR = 1.31, 95% CI = 1.01–1.71, *p* ≤ 0.05).

**Conclusions:**

Our data confirm the heterogeneity of breast tumors at a functional proteomic level and dissects the relationship between metabolism-related proteins, pathological features and patient survival. These observations highlight the importance of SHMT2 and ASCT2 as valuable individual prognostic markers and potential targets for personalized breast cancer therapy.

**Trial registration:**

ClinicalTrials.gov, NCT01592825. Registered on 3 May 2012.

**Electronic supplementary material:**

The online version of this article (doi:10.1186/s13058-017-0905-7) contains supplementary material, which is available to authorized users.

## Background

Worldwide, breast cancer (BC) is the most prevalent cancer entity among women and is known as a heterogeneous disease in terms of tumor morphology and molecular structure [1–4]. Although many genes and proteins have been investigated as prognostic and predictive factors, only a few are decisive for treatment. This is reflected in the classicl breast cancer stratification into receptor-defined subtypes, termed luminal A-like, luminal B-like, triple negative breast cancer (TNBC), and human epidermal growth factor receptor 2 (HER2)-positive, as common clinical practice [5, 6]. However, expanding protein profiling towards novel directions could provide new insights into molecular mechanisms associated with the observed heterogeneous clinical outcome. Moreover, analyzing these protein profiles harbors the potential for identification of prognostic markers and druggable targets off the beaten track.

Altered metabolism has long been known to characterize tumors ever since Otto Warburg reported his first observations of the metabolic changes that accompany malignancy [7]. Furthermore, deregulated cancer metabolism has regained attention and is regarded as a new hallmark of cancer [8]. Metabolic transformations have been intensively studied over recent years and as a result, the first strategies to target the altered metabolism of cancer cells are emerging [9].

Mutations in metabolic enzymes can drive tumorigenesis; more often, however, cancer metabolism is transformed by altered abundance and activity of metabolic enzymes [10]. Proliferative cells alter their metabolism to support biosynthetic reactions required for accumulation of biomass and the production of macromolecules [11]. Reprogrammed cellular metabolism involves increased glucose intake and glutamine addiction. Glutamine is the most abundant amino acid in serum and represents a fundamental source for nucleotide and amino acid synthesis. “Glutamine addiction”, which is characterized by poor cancer cell survival in the absence of glutamine, has been observed in several cancer entities [12]. Glutamine acts as a nitrogen donor for nucleotide and protein synthesis, and is converted via glutaminase to glutamate, which represents the main nitrogen donor for the synthesis of nonessential amino acids [13]. Furthermore, glutamine has been described as an essential activator of the mammalian target of rapamycin complex 1 (mTORC1), which regulates protein translation, cell growth and autophagy [14]. Glutamine is transported by several families of amino acid transporters, of which ASC amino-acid transporter 2 (ASCT2), also named solute carrier family 1 member 5 (SLC1A5), belongs to the most ubiquitously expressed glutamine transporters in human cancer cells [15]. Apart from glutamine metabolism, serine and glycine metabolism are also important mediators in cancer cell development. Serine and glycine are biosynthetically linked, and together provide essential precursors for the synthesis of proteins, nucleic acids, and lipids that are crucial to cancer cell growth. Serine hydroxymethyltransferase (SHMT) reversibly converts serine to glycine, connecting the serine and glycine pathways. Glycine is required to maintain the cellular redox balance and also sustains oxidative phosphorylation in the mitochondria [16]. It has been shown that glycine uptake and catabolism are able to promote tumorigenesis and malignancy, suggesting that serine and glycine metabolism could be a target for therapeutic intervention [17].

Nevertheless, the criteria used to evaluate tumor metabolism are still not well-established and thus are not universally applied. Also, it is unclear mechanistically how metabolic characteristics of the tumor influence patient outcome and how they can be utilized in the clinical management of tumors. Therefore, it is necessary to obtain a better understanding of molecular mechanisms underlying the heterogeneity of breast cancer metabolism. Transcriptional profiling of genes associated with cancer metabolism has to some extent identified associations with different clinical features [18]. However, the breast cancer transcriptome does not directly translate into proteome and comprehensive analysis of messenger RNA (mRNA) expression does not reflect all layers of biological complexity [19, 20]. Thus, a systematic study of protein expression profiles related to major metabolic pathways may facilitate a more precise classification and exploration of prognostic markers in breast cancer.

During recent years, reverse phase protein array (RPPA) has emerged as a powerful high-throughput approach for targeted proteomics [21, 22]. RPPA allows the quantification of protein expression profiles in large sample sets while requiring very low amounts of biological sample. Therefore, the RPPA platform is ideally suited for the analysis of clinical materials and biomarker discovery purposes [23–25].

In respect of the current focus on precision medicine, the identification of novel therapeutic proteins and prognostic biomarkers is critical for future clinical drug discovery and patient stratification purposes. The objective of this study was to assess the relationship between protein profiles of major metabolic pathways and their prognostic value in patients with breast cancer treated in accordance to national guidelines. We applied RPPA-based functional proteomics to a large number of patient samples from a multicenter prospective cohort. We assessed clusters of breast cancer subgroups based on metabolism-associated protein expressions. Furthermore, we aimed to identify new markers and prognostic factors associated with patient outcome.

## Methods

### Patients and tissue samples

Human primary breast cancer samples were collected at the Martin-Luther University, Halle-Wittenberg between 2009 and 2011 as part of the multicenter prospective PiA trial (NCT 01592825). Only fresh frozen tissue samples from female patients with operable non-metastasized breast cancer were included. The study was approved by the ethics committee of the Martin-Luther University Halle-Wittenberg and informed consent was obtained from each patient. A cohort of 801 primary tumor tissue samples was investigated using RPPA. Tumor specimens were fresh frozen after surgery and stored at −80 °C until further use. Tumor content was verified by histopathological assessment. Clinicopathological parameters were obtained for each patient and documented using SPSS 22, SPSS Inc., Chicago, IL, USA. The TNM staging system was used [26]. Patient information was anonymized prior to analysis. Receptor-defined breast cancer subtypes were determined according to the St. Gallen classification [27]. Due to missing Ki-67 values, we used histopathological grading to assess cell proliferation [28]. The following stratification system was applied:

- Luminal A-like: estrogen receptor (ER) positive and/or progesterone receptor (PgR) positive, HER2 negative, grade 1 or 2.
- Luminal B-like (HER2 negative): ER positive and/or PgR positive, HER2 negative, grade 3.
- Luminal B-like (HER2 positive): ER positive and/or PgR positive, HER2 positive, all grades.
- HER2 positive (non-luminal-like): ER negative and PgR negative, HER2 positive, all grades.
- Triple negative breast cancer (TNBC): ER negative, PgR negative, HER2 negative, all grades.

The standardized definitions for efficacy endpoints (STEEP) criteria were used as endpoint definitions [29]. Additional information on patient and tumor characteristics are illustrated in Table 1.

**Table 1:** Patient and tumor characteristics

	Total	Percentage
Number of patient samples		
Total	801	100
Age		
Mean ± SD	62.25 ± 13.7	
Median (range)	63 (22–90)	
Tumor size		
<2 cm	400	49.9
≥2–5 cm	358	44.7
>5 cm	43	5.4
Histology		
Ductal	638	79.7
Lobular	118	14.7
Other	45	5.6
Tumor stage		
T1	413	51.6
T2	342	42.7
T3	38	4.7
T4	8	1
Grade		
I	91	11.4
II	502	62.7
III	208	26
Nodal status		
N0	492	61.4
N1	226	28.2
N2	51	6.4
N3	32	4
Menopausal status		
Premenopausal	167	20.8
Perimenopausal	51	6.4
Postmenopausal	583	72.8
Receptor status		
ER+	681	85
ER-	120	15
PgR+	563	70.3
PgR-	238	29.7
HER2+	110	13.7
HER2-	691	86.3
HR+	688	85.9
HR-	113	14.1
Receptor-defined subtype		
Luminal A-like	510	63.7
Luminal B-like (HER2 positive)	74	9.2
Luminal B-like (HER2 negative)	104	13
HER2 positive (non-luminal-like)	36	4.5
TNBC	77	9.6

*ER* estrogen receptor, *PgR* progesterone receptor, *HER2* human epidermal growth factor receptor 2, *TNBC* triple negative breast cancer

### Reverse phase protein array profiling

Frozen tumor specimens were homogenized using a bead mill and tissue protein extraction reagent (50 mM Tris, pH 8.5, 138 mM NaCl, 2.7 mM KCl, 1% Triton X-100). Total protein concentration was determined by bicinchoninic acid protein assay (Thermo Scientific). Tumor lysates were adjusted to a total protein concentration of 2 μg/μl, mixed with 4 × SDS sample buffer (10% glycerol, 4% SDS, 10 mM DTT, 125 mM Tris–HCl, pH 6.8) and denaturated at 95 °C for 5 min. Protein lysates and dilution series of tumor sample pools serving as controls, were spotted as technical triplicates on nitrocellulose coated glass slides (Oncyte Avid, Grace-Biolabs) using an Aushon 2470 contact spotter (Aushon BioSystems). Post spotting, slides were incubated with blocking buffer (Rockland Immunochemicals) in TBS (50%, v/v) containing 5 mM NaF and 1 mM Na3VO4 for 2 h at room temperature. Incubation with target-specific primary antibodies was applied at 4 °C overnight. Primary antibodies were selected to cover a range of metabolic pathways and to achieve a broad perspective on breast cancer metabolism (Additional file 7: Table S5). Antibody validation was carried out as previously described [30]. Primary antibodies were detected with Alexa Fluor 680 F(ab')2 fragments of goat anti-mouse IgG or anti-rabbit IgG (Life Technologies) in 1:12000 dilution. In addition, representative slides were stained for total protein quantification using Fast Green FCF protein dye as described before [31]. TIFF images of all slides were obtained at an excitation wavelength of 685 nm and at a resolution of 21 μm using an Odyssey Scanner (LI-COR, Biosciences). Signal intensities of individual spots were quantified using GenePixPro 7.0 (Molecular Services Inc.). Data preprocessing and quality control were performed using the *RPPanalyzer* R-package [32].

### Immunohistochemical analysis

Immunohistochemical analyses (IHC) were performed on 4-μm tissue sections. Protein expression was assessed using Bond Max Polymer Refine Immunohistochemistry protocol. Primary antibodies were diluted 1:250. Epitope retrieval was performed with Bond Epitope Retrieval Solution for 30 min at pH6, followed by a peroxidase block. Primary antibody was incubated for 20 min and detected using Bond Polymer Refine Detection with 3,3-diaminobenzidine (DAB) substrate. IHC was performed by a pathologist as a semi-quantitative visual score, based on the fraction of cytoplasmic staining above background.

### Statistical and bioinformatic analyses

#### Hierarchical clustering

Hierarchical cluster analysis was performed on *z* scores of protein expression levels using Ward's minimum variance method and squared Euclidean distance. Patient samples and protein targets were clustered simultaneously and the resulting dendrograms were visualized with a heatmap depicting *z* score values. *RPPanalyzer* R-package was used for visualization, with adjustment of color bars according to the clinicopathological features of interest and exploiting the *dendextend* R-package for dendrogram color-coding [32, 33].

#### Univariate analysis

The relationship between clinicopathological variables and the three patient clusters was evaluated using analysis of variance (ANOVA), the Kruskal-Wallis rank sum test, and Fisher's exact test, as appropriate. The relationship between the variables and the patient groups, stratified based on the median expression of a protein, was evaluated using the *t* test, Wilcoxon rank sum test, and Fisher's exact test, as appropriate.

#### Survival analysis

Kaplan-Meier analysis of overall survival (OS) and recurrence-free survival (RFS) was performed on patients stratified into groups (based on receptor-defined subtypes, median expression level or patient dendrogram clusters). The difference in Kaplan-Meier curves was tested using the log-rank test implemented in the *survival* R-package [34]. Univariate Cox proportional hazard regression models were applied to test individual protein target association with OS and RFS [35]. For each target the exponent of the estimated regression coefficient is reported as a hazard ratio (HR) with its 95% confidence intervals (CI). *P* values were adjusted for multiple testing resulting in false-discovery rate (FDR) values [36]. Univariate Cox proportional hazard regression models were further used to evaluate clinicopathological variables. Multivariate Cox analyses were then performed on selected non-correlated clinicopathological covariates for each of the proteins that was significant in the univariate Cox analysis.

#### STRING visualization

The Search Tool for the Retrieval of Interacting Genes/Proteins (STRING) database (Version 10) of the STRING Consortium was used for visualization of protein interactions by choosing Gene IDs corresponding to proteins [37]. If not otherwise stated, the data were analyzed using the R statistical computing environment (version 3.0.2) [38]. A *p* value <0.05 was considered statistically significant. The complete RPPA data matrix with corresponding clinicopathological parameters is appended in Additional file 8: Table S6.

## Results

### Unsupervised clustering of protein expression profiles in patients with breast cancer

**Fig. 1:**
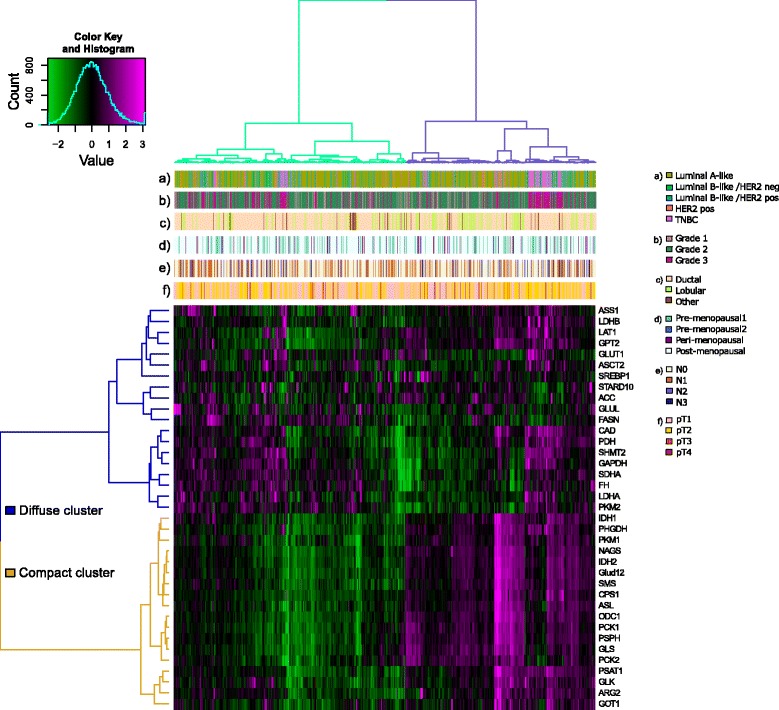
Unsupervised clustering of protein profiles. The heatmap represents expression levels of 37 metabolism related proteins after unsupervised hierarchical clustering. The dataset consists of 801 tumor specimen. The *z* scores of log2 transformed protein expression levels are color-coded on a low-to-high scale (green-black-magenta). Dendrogram branches divide the patient set into a green and violet cluster and protein targets into a “diffuse” and “compact” cluster. Annotation bars include receptor-defined subtypes (**a**), histological grade (**b**), histology (**c**), menopausal status (**d**), nodal status (**e**) and T stage (**f**). HER2, human epidermal growth factor receptor 2; TNBC, triple negative breast cancer

To investigate altered expression patterns of metabolism-related proteins in tumorigenesis of BC, we performed RPPA of 801 patient specimens. The clinicopathological features of the cohort are summarized in Table 1. The median follow up of the cohort was 55.44 months for overall survival (OS) and 54.46 months for recurrence-free survival (RFS). In a first step, the patient profiles of 37 metabolism-related proteins were assessed by unsupervised hierarchical clustering. As illustrated in Fig. 1, clustering divided the cohort into two patient subgroups (green, *n* = 440; violet, *n* = 361).

To elucidate the potential association with survival in the two subgroups, we performed Kaplan-Meier analysis (Additional file 1: Figure S1). We observed no significant association with OS or RFS. However, a distinct horizontal partition seemed to be a more dominant feature of the heatmap. A separation into two protein expression subgroups indicated functional differences throughout the whole patient cohort. Therefore, we divided the protein dendrogram into two protein subgroups, a “diffuse”’ cluster (blue, *n* = 19), characterized by a heterogeneous protein expression pattern and a “compact” cluster (gold, *n* = 18) with a clear protein expression pattern. Notably, the impact of the compact protein cluster in driving the initial clustering and formation of the two patient clusters, seemed to subdue the effects of the diffuse cluster. Therefore, we focused on re-investigating the diffuse protein cluster separately.

### Diffuse protein signature revealed three patient clusters significantly associated with survival

**Fig. 2:**
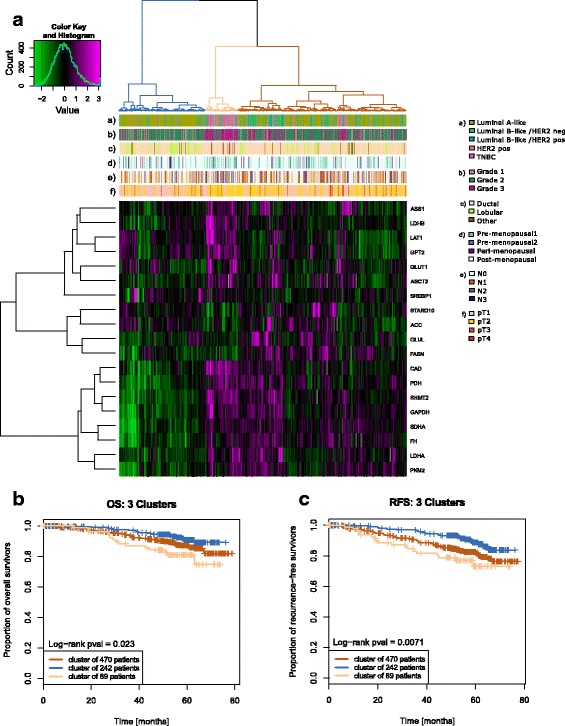
Unsupervised clustering and analyses based on “diffuse” cluster refinement (**a**). The heatmap represents metabolism-related protein expression levels of the diffuse target signature after unsupervised hierarchical clustering of 801 tumor specimen. The *z* scores of log2-transformed protein expression levels are color-coded on a low-to-high scale (green-black-magenta). Annotation bars include receptor-defined subtypes (a), histological grade (b), histology (c), menopausal status (d), nodal status (e) and T stage (f). Statistical analysis of the three patient clusters (blue, yellow, brown) is shown in Additional file 3: Table S1. Kaplan-Meier curves represent the proportion of overall survivors (**b**) and recurrence-free survivors (**c**), compared by log-rank test

Hierarchical clustering of the 19 protein targets representing the diffuse cluster, resulted in three refined patient clusters based on the dendrogram arrangement (Fig. 2a).

The blue (*n* = 242), yellow (*n* = 89) and brown cluster (*n* = 470) were compared in survival analysis of OS and RFS. This revealed a significant difference among the clusters in both OS (*p* = 0.023, Fig. 2b) and RFS (*p* = 0.0071, Fig. 2c). The blue cluster showed the most favorable OS and RFS, whereas the yellow cluster represented the worst. Clinical parameters (age, tumor size, histology, T stage, grade, node status, menopausal status and receptor-defined status) were examined for differences in distribution between the patient clusters (Additional file 3: Table S1). Univariate comparison across the patients’ groups showed that all clinical parameters were significantly different between the three clusters (*p* ≤ 0.05). Furthermore, multivariate analysis was conducted based on selected clinicopathological covariates and while multivariate analysis became null once additional clinical covariates were included, univariate associations between the clusters and OS/RFS were significant.

### The proteomic network of the diffuse and compact cluster

To visualize the biological context of the proteins representing the diffuse and compact clusters at a glance, we visualized them in two protein networks by using the STRING database (Additional file 2: Figure S2). As part of the diffuse cluster, we observed all proteins related to glycine synthesis (SHMT2), lipid and fatty acid synthesis (FASN, STARD10, ACC and SREBF1), and glycolysis and lactate production (GLUT1, GAPDH, PKM2, LDHA and LDHB). The compact cluster in comparison is composed of all measured proteins associated with serine synthesis (PHGDH, PSAT1 and PSPH). Pyruvate kinase isozyme M1 (PKM1) was the only glycolysis protein represented in the compact cluster. Proteins related to the tricarboxylic acid (TCA) cycle, urea cycle and glutaminolysis, were found in both the diffuse and the compact cluster.

### Correlation between individual target expression and clinicopathological characteristics

In order to identify individual proteins responsible for the survival association of the diffuse cluster, and to evaluate their potential role as biomarkers, we next analyzed the expression of all probed proteins individually. The association between each protein expression level and OS and RFS was tested using univariate Cox proportional hazard regression models and protein expression was treated as a continuous variable (Additional file 4: Table S2). Out of 37 metabolism-related proteins tested, SHMT2 and ASCT2 were found to be significantly associated with OS (Table 2). Univariate Cox analysis of RFS identified 6 out of 37 proteins to be significantly associated with outcome (Table 3).

**Table 2:** Protein targets significantly associated with overall survival (OS)

Target	HR	95% CI	*P* value	FDR	Affiliation
SHMT2	1.93	1.48–2.51	<0.001	<0.001	Serine metabolism
ASCT2	1.83	1.39–2.42	<0.001	<0.001	Glutamine metabolism

OS events = 83

*HR* hazard ratio, *FDR* false-discovery rate, *CI* confidence interval, *SHMT2* serine hydroxymethyltransferase 2, *ASCT2* ASC amino-acid transporter 2

**Table 3:** Protein targets significantly associated with recurrence-free survival (RFS)

Target	HR	95% CI	*P* value	FDR	Affiliation
SHMT2	1.88	1.50–2.36	<0.001	<0.001	Serine metabolism
ASCT2	1.83	1.45–2.31	<0.001	<0.001	Glutamine metabolism
GAPDH	1.52	1.19–1.94	<0.001	0.009	Glucose metabolism
FH	1.65	1.20–2.27	0.002	0.019	TCA cycle
CAD	2.07	1.29–3.33	0.003	0.019	Pyrimidine metabolism
PKM2	1.46	1.13–1.88	0.003	0.02	Glucose metabolism

RFS events = 109

*HR* hazard ratio, *FDR* false-discovery rate, *CI* confidence interval, *SHMT2* serine hydroxymethyltransferase 2, *ASCT2* ASC amino-acid transporter 2, *GAPDH* glyceraldehyde-3-phosphate dehydrogenase, *TCA* tricarboxylic acid, *FH* fumarate hydratase, *CAD* carbamoyl-phosphate synthetase 2, *PKM2* pyruvate kinase 2

Patients with breast cancer were further stratified into “low” and “high” protein expression groups to explore the relationship with clinicopathological variables. This was based on the median protein expression of SHMT2, ASCT2, GAPDH, FH, CAD and PKM2 (Additional file 5: Table S3). Univariate analysis showed that all six protein expression profiles were significantly associated with tumor size, T stage, grade, nodal status and receptor-defined subgroups. Except for CAD, all proteins showed significant association with histology results, whereas PKM2 protein expression was the only protein profile significantly correlated with age. No significant difference between the protein expression profiles and menopausal status was observed.

### SHMT2 and ASCT2 protein expression as independent prognostic factors in patients with breast cancer

To further confirm our findings, multivariate Cox analyses for OS and RFS was conducted based on selected clinicopathological covariates and univariate significance. Proteins that were significant in the univariate Cox analysis were included (Additional file 6: Table S4).

**Fig. 3:**
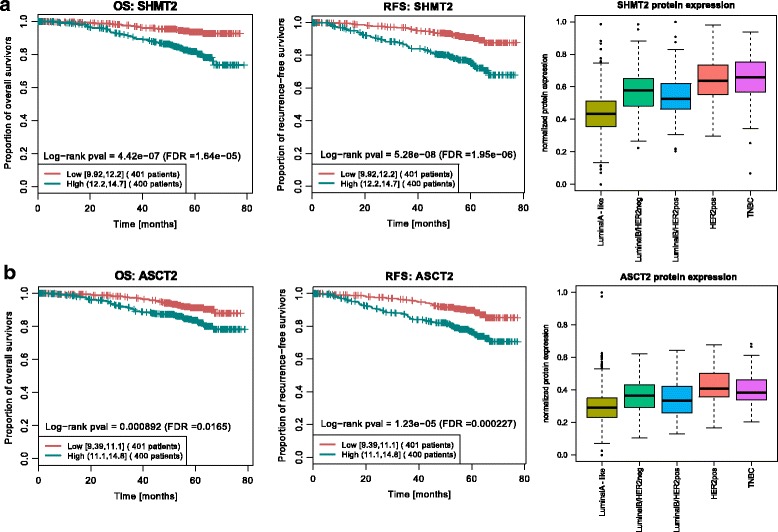
Kaplan-Meier survival estimates and boxplot representation of key targets associated with survival. Kaplan-Meier plots of serine hydroxymethyltransferase 2 (SHMT2) and ASC amino-acid transporter 2 (ASCT2) for overall survival (OS) (**a**), and recurrence-free survival (RFS) (**b**). Statistical difference in outcome between high (*n* = 400) and low (*n* = 401) expression were compared by log-rank test. Boxplots represent the relative target protein expression per receptor-defined subtype, luminal A-like (*n* = 510), luminal B-like human epidermal growth factor receptor 2-negative (HER2-neg) (*n* = 104), luminal B-like HER2-positive (HER2-pos) (*n* = 74), HER2-pos (*n* = 36), triple negative breast cancer (TNBC) (*n* = 77). FDR, false discovery rate; pval, *p* value

To address whether SHMT2 and ASCT2 protein expression are independent prognosticators for OS and RFS, we analyzed the association between SHMT2/ASCT2 protein expression levels and clinical characteristics of BC, using multivariate Cox models. This revealed that high SHMT2 protein expression is an independent negative prognostic factor for OS (*p* = 0.011; Table 4) and both high SHMT2 and high ASCT2 protein expression levels are independent negative prognostic factors for RFS (SHMT2, *p* = 0.003; ASCT2, *p* = 0.042; Table 5) in patients with BC. Kaplan-Meier survival estimates, based on dichotomized protein expression data, subsequently confirmed that patients with BC with high SHMT2 and high ASCT2 protein expression had significantly shorter OS (SHMT2, *p* < 0.001; ASCT2, *p* = 0.0165) and RFS (SHMT2, *p* < 0.001; ASCT2, *p* < 0.001), (Fig. 3).

**Table 4:** Univariate and multivariate Cox regression analysis of overall survival

Characteristics	Univariate analysis	SHMT2		ASCT2	
		Multivariate analysis		Multivariate analysis	
	*P* value	Hazard ratio (95% CI)	*P* value	Hazard ratio (95% CI)	*P* value
Protein expression high vs. low		1.53 (1.10–2.12)	0.011	1.23 (0.90–1.68)	0.194
Age at surgery (years)	<0.001	1.06 (1.03–1.09)	<0.001	1.06 (1.03–1.08)	<0.001
Tumor size	<0.001	*Not included*		*Not included*	
< 2 cm					
≥2–-5 cm					
>5 cm					
Histology	0.306	*Not included*		*Not included*	
Ductal vs. non-ductal					
T stage	<0.001				
T1 vs. ≥T2		1.46 (0.88–2.40)	0.141	1.49 (0.90–2.47)	0.123
Grade	<0.001				
I		*Reference*		*Reference*	
II		1.69 (0.52–5.49)	0.385	1.77 (0.54–5.76)	0.345
III		2.40 (0.70–8.23)	0.163	2.95 (0.87–9.99)	0.081
Nodal status	<0.001				
N0 vs. ≥N1		1.86 (1.18–2.92)	0.007	1.85 (1.17–2.92)	0.008
Menopausal status	0.001				
Pre/peri vs. postmenopausal		0.72 (0.28–1.85)	0.489	0.80 (0.31–2.05)	0.64
Receptor status					
ER- vs. ER+	<0.001	*Not included*		*Not included*	
PgR- vs. PgR+	<0.001	*Not included*		*Not included*	
HER2- vs. HER2+	0.682	*Not included*		*Not included*	
HR- vs. HR+	<0.001	0.72 (0.42–1.22)	0.217	0.63 (0.37–1.06)	0.082
Receptor-defined subtypes	<0.001	*Not included*		*Not included*	
Luminal A-like					
Luminal B-like (HER2 positive)					
Luminal B-like (HER2 negative)					
HER2 positive (non-luminal-like)					
TNBC					

*SHMT2* serine hydroxymethyltransferase 2, *ASCT2* ASC amino-acid transporter 2, *CI* confidence interval, *Pre-peri* premenopausal/perimenopausal, *ER* estrogen receptor, *PgR* progesterone receptor, *HER2* human epidermal growth factor receptor 2, TNBC triple negative breast cancer

A *p* value <0.05 was considered statistically significant

**Table 5:** Univariate and multivariate Cox regression analysis of recurrence-free survival

Characteristics	Univariate analysis	SHMT2		ASCT2	
		Multivariate analysis		Multivariate analysis	
	*P* value	Hazard ratio (95% CI)	*P* value	Hazard ratio (95% CI)	*P* value
Protein expression high vs. low		1.54 (1.16–2.04)	0.003	1.31 (1.01–1.71)	0.042
Age at surgery (years)	<0.001	1.04 (1.02–-1.07)	<0.001	1.04 (1.02–1.06)	<0.001
Tumor size	<0.001	*Not included*		*Not included*	
<2 cm					
≥ 2–5 cm					
>5 cm					
Histology	0.11	*Not included*		*Not included*	
Ductal vs. non-ductal					
T stage	<0.001				
T1 vs. ≥T2		1.77 (1.15–2.74)	0.01	1.80 (1.16–2.80)	0.009
Grade	<0.001				
I		*Reference*		*Reference*	
II		1.79 (0.65–4.98)	0.262	1.85 (0.66–5.14)	0.24
III		2.18 (0.75–6.35)	0.154	2.64 (0.92–7.59)	0.072
Nodal status	<0.001				
N0 vs. ≥N1		1.62 (1.10–2.40)	0.015	1.59 (1.07–2.35)	0.021
Menopausal status	0.01				
Pre/peri vs. postmenopausal		0.65 (0.31–1.38)	0.263	0.73 (0.35–1.54)	0.41
Receptor status					
ER- vs. ER+	<0.001	*Not included*		*Not included*	
PgR- vs. PgR+	<0.001	*Not included*		*Not included*	
HER2- vs. HER2+	0.489	*Not included*		*Not included*	
HR- vs. HR+	<0.001	0.79 (0.49–1.27)	0.334	0.69 (0.43–1.10)	0.115
Receptor-defined subtypes	<0.001	*Not included*		*Not included*	
Luminal A-like					
Luminal B-like (HER2 positive)					
Luminal B-like (HER2 negative)					
HER2 positive (non-luminal-like)					
TNBC					

*SHMT2* serine hydroxymethyltransferase 2, *ASCT2* ASC amino-acid transporter 2, *CI* confidence interval, *Pre-peri* premenopausal/perimenopausal, *ER* estrogen receptor, *PgR* progesterone receptor, *HER2* human epidermal growth factor receptor 2, TNBC triple negative breast cancer

A *p value* <0.05 was considered statistically significant

**Fig. 4:**
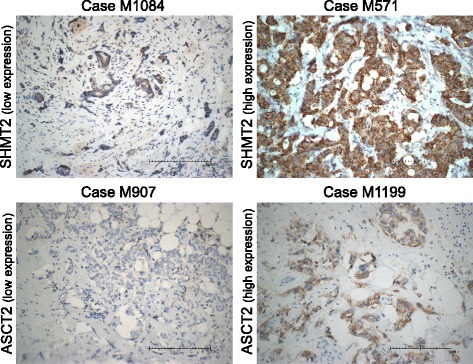
Representative immunoexpression of serine hydroxymethyltransferase 2 (SHMT2) and ASC amino-acid transporter 2 (ASCT2). Cases were selected on the basis of reverse phase protein array (RPPA) protein expression results. Representative pictures of the highest or lowest 10% of cases based on the target expression over all cases. SHMT2 immunoexpression is elevated in Case M571 and low in Case M1084. ASCT2 immunoexpression is elevated in Case M1199 and low in Case M907. The scale bar indicates 200 μm (×20)

We additionally explored the distribution of SHMT2 and ASCT2 protein expression across BC subtypes. This identified higher protein expression of both targets in the aggressive HER2-positive and the TNBC breast cancer subtype, in comparison to the luminal subgroup (Fig. 3). SHMT2 and ASCT2 immunostaining of representative cases were selected on the basis of RPPA protein expression analysis and revealed a confirming pattern of cellular target protein expression in RPPA vs. IHC. Cases of high target-protein expression in RPPA also represented a high cellular target protein expression in IHC and vice versa (Fig. 4). Taken together, these results illustrate the prognostic value of profiling proteome data and highlight the importance of including the proteomic level in biomarker research.

## Discussion

Since breast tumors are heterogeneous at the molecular level and in outcome, future clinical management should include personalized tumor characterization, which leads to improved treatment decisions. So far, the metabolic state of tumors has not been studied widely and is insufficiently embodied by current molecular biomarkers that predict adverse clinical outcome. Moreover, large cohort studies have addressed metabolic biomarkers at the genomic level only. Even though genomic information does translate to a certain extent into phenotypic characteristics, genomic and transcriptomic screens of tumors in patients with BC have proven unsuccessful in predicting protein status [39, 40]. Therefore, a complementary study investigating the metabolic landscape of breast cancer at the proteomic level is beneficial in identifying proteome-based biomarkers with clinical impact.

In this study, we used RPPA to generate protein expression data from 801 breast tumor specimens, in order to identify prognostic markers and to gain insights into the metabolic heterogeneity of breast cancer. Clustering analysis of the whole dataset separated the metabolism associated proteins into a diffuse and a compact subgroup, indicating different metabolic profiles. STRING visualization of the protein distribution depicted a prominent role of glycolysis towards lactate production in the diffuse cluster. Also, SHMT2, primarily responsible for synthesizing glycine from serine, was a part of the diffuse cluster, whereas all proteins of the serine pathway (PHGDH, PSAT1 and PSPH) were distributed in the compact cluster. Interestingly, key enzymes of the TCA cycle, crucial for citrate production, like FH and SDHA, were present in the diffuse cluster, whereas IDH1 and IDH2, which mainly drive the TCA cycle towards α-ketoglutarate production, were part of the compact cluster. This observation was supported by the presence of PDH, STARD10 and FASN and hints towards distinct citrate production in order to fuel the lipid and fatty acid synthesis. Notably, the glutamine transporters ASCT2 (SLC1A5) and SLC7A5, and the glutamine producing enzyme GLUL, were also part of the diffuse cluster. Altogether, the protein composition of the diffuse cluster hints towards glucose consumption, glutamine addiction and glycine production and indicates a more active Warburg-like characteristic in comparison to the compact cluster [41].

Subsequent clustering analysis based on the diffuse-cluster protein levels identified three patient clusters, which are significantly associated with survival on univariate analysis. These three patient clusters do not fully reflect the receptor-defined subtypes and may thus provide additional information towards understanding breast cancer heterogeneity. Interestingly, further analysis showed that all proteins found to be significantly correlated with survival, were part of the diffuse cluster. SHMT2, ASCT2, GAPDH, FH, CAD and PKM2 were associated with RSF and SHMT2 on univariate analysis, and ASCT2 was associated with with OS. Further, we explored the biomarker potential of these proteins in multivariate Cox analyses.

Multivariate analysis identified SHMT2 and ASCT2 protein expression levels as significantly associated with age, nodal status and T stage (RFS only). Moreover, high SHMT2 protein levels were significantly associated with poor RFS and OS. High protein expression of ASCT2 was significantly correlated with poor RFS. Patients of HER2-positive and HR-negative breast cancer had increased SHMT2 and ASCT2 levels compared to patients with luminal-like cancer. Notably, the significant correlation between ASCT2 or SHMT2 and nodal status, T stage and survival indicates a connection of higher metabolic activity and associated protein expression in metastatic tumors and tumors with further progression. These observations are in line with studies reporting that the metabolic demands of cancer cells are related to their cell size, progression and protein synthesis rates [42].

Glutamine metabolism is considered to be a therapeutic target, as some cancer cells exhibit high uptake of and addiction to this non-essential amino acid [41]. Recent studies have demonstrated that the primary glutamine transporter, ASCT2, can promote tumor cell survival, growth and cell cycle progression in neuroblastoma, colorectal cancer, prostate cancer, clear-cell renal cell carcinoma and non-small cell lung cancer [43–47]. The ubiquitous tissue expression, along with its ability to transport crucial amino acids, indicates the central role of ASCT2 in physiological processes including glutamine homeostasis, embryogenesis and retroviral infection [48, 49]. Glutamine is not only an important nutrient for cancer cell survival, but also a crucial mediator for immune cell functions. ASCT2 was shown to be involved in inflammatory T cell responses, which might exert key functions in tumor immunity [50]. Besides its significance in prognosis, ASCT2 has also gained more attention in cancer treatment during recent years. ASCT2 is considered to be a major regulator of glutamine metabolism and thus represents an important regulator of cancer development [51]. ASCT2 also regulates the cellular nutrient uptake and concentration [52, 53]. Several studies indicate that blocking glutamine uptake might be an attractive strategy for cancer therapy. We showed that high protein levels of ASCT2 are correlated with unfavorable prognosis. Blocking the glutamine uptake by utilizing ASCT2 as a potential therapeutic target and reducing its protein expression, could therefore be a promising approach.

Besides glutamine, serine and glycine metabolism is also crucial in cancer cell development. Serine and glycine are biosynthetically linked, and besides cancer growth also affect the cellular antioxidative capacity, thus supporting tumor homeostasis. SHMT2 has been implicated as an essential factor in serine and glycine metabolism in several cancer cell types, including breast cancer [17]. SHMT2 catalyzes the reversible reaction of serine and tetrahydrofolate to glycine and 5,10-methylene tetrahydrofolate. Genomic studies have shown that high levels of glycine are associated with poor prognosis in breast cancer, irrespective of the ER status [54]. We could demonstrate that high protein levels of SHMT2 are correlated with poor outcome. Inhibition of glycine synthesis by reducing SHMT2 protein expression could therefore represent a promising strategy to employ SHMT2 as a potential therapeutic target. Considering the metabolic heterogeneity of breast cancer, SHMT2 and ASCT2 might be useful as potential markers in risk stratification and targets for drug development. Notably, to date there are no SHMT2 and ASCT2 inhibitors commercially available for cancer therapy. To our knowledge this is the first study to report the prognostic value of SHMT2 and ASCT2 at the protein expression level in patients with breast cancer.

Although our study revealed the clinical significance of SHMT2 and ASCT2 in breast cancer, some limitations warrant further investigation. For instance, the molecular mechanisms and functional behavior of SHMT2 and ASCT2 in breast cancer merit further exploration. Furthermore, investigations in an independent external cohort are needed to validate our findings. We seek to further investigate the mechanisms discussed in future studies and will conduct long-term follow up of the patient cohort to monitor the prognostic power of our results.

## Conclusions

In this newly generated breast cancer dataset, we identified metabolism-associated proteins linked to breast cancer progression. We found metabolic clusters of breast cancer, characterized by differences at the proteomic level. Particularly, proteins mapping to the diffuse cluster, were found to be associated with poor prognosis. Univariate and multivariate analyses supported the crucial role of SHMT2 and ASCT2 protein expression as independent prognostic factors in breast cancer. High protein expression of SHMT2 and ASCT2 were significantly associated with shorter RFS. Moreover, high SHMT2 protein expression was also a predictor for shorter OS. In summary, SHMT2 and ASCT2 protein expression were identified as novel potential prognostic biomarkers for patients with breast cancer, as their high protein expression is associated with poor outcome.

## Additional files

Additional file 1: Figure S1. Kaplan-Meier analysis of green and violet cluster. Kaplan-Meier curves show proportions of overall survivors (OS) and recurrence-free survivors (RFS) of two separate clusters. Statistical difference in outcome between Kaplan-Meier curves were compared by log-rank test. (PDF 93 kb)

Additional file 2: Figure S2. Protein network visualization. STRING illustrations are based on proteins represented in the “compact”’ cluster subgroup (**A**) and proteins represented in the “diffuse” cluster subgroup (**B**). STRING visualization was performed for each group individually and the evidence based network edges were set to an interaction score of 0.4. The given legend shows the type of interactions that were selected for the visualization. (PDF 2496 kb)

Additional file 3: Table S1. Relationship between clusters and clinical and pathological characteristics. (XLS 36 kb)

Additional file 4: Table S2. Univariate Cox proportional hazard regression models of OS and RFS. (XLS 33 kb)

Additional file 5: Table S3. Correlation between key target expression and patients and tumor characteristics. (XLS 46 kb)

Additional file 6: Table S4. Univariate and multivariate Cox regression analyses of OS and RFS. (XLS 37 kb)

Additional file 7: Table S5. List of antibodies used for RPPA-based profiling. (XLS 30 kb)

Additional file 8: Table S6. RPPA data expression matrix with matched clinical data. (XLS 749 kb)

## Abbreviations

ASCT2: ASC amino-acid transporter 2
BC: Breast cancer
CI: Confidence interval
ER: Estrogen receptor
FDR: False-discovery rate
GAPDH: Glyceraldehyde-3-phosphate dehydrogenase
HER2: Human epidermal growth factor receptor 2
HR: Hazard ratio
HR-: Hormone receptor negative
HR+: Hormone receptor positive
OS: Overall survival
PgR: Progesterone receptor
RFS: Recurrence-free survival
RPPA: Reverse phase protein array
SHMT2: Serine hydroxymethyltransferase 2
STEEP: Standardized definitions for efficacy end points
STRING: Search Tool for the Retrieval of Interacting Genes/Proteins
TCA: Tricarboxylic acid
TCGA: The Cancer Genome Atlas
TNBC: Triple negative breast cancer

## References

[1] Ferlay J, Soerjomataram I, Dikshit R, Eser S, Mathers C, Rebelo M (2015). Cancer incidence and mortality worldwide: sources, methods and major patterns in GLOBOCAN 2012. Int J Cancer.

[2] Curtis C, Shah SP, Chin SF, Turashvili G, Rueda OM, Dunning MJ (2012). The genomic and transcriptomic architecture of 2,000 breast tumours reveals novel subgroups. Nature.

[3] Cancer Genome Atlas N (2012). Comprehensive molecular portraits of human breast tumours. Nature.

[4] Weigelt B, Reis-Filho JS (2009). Histological and molecular types of breast cancer: is there a unifying taxonomy?. Nat Rev Clin Oncol.

[5] Goldhirsch A, Winer EP, Coates AS, Gelber RD, Piccart-Gebhart M, Thurlimann B (2013). Personalizing the treatment of women with early breast cancer: highlights of the St Gallen International Expert Consensus on the Primary Therapy of Early Breast Cancer 2013. Ann Oncol.

[6] Sotiriou C, Pusztai L (2009). Gene-expression signatures in breast cancer. N Engl J Med.

[7] Warburg O (1956). On the origin of cancer cells. Science.

[8] Hanahan D, Weinberg RA (2011). Hallmarks of cancer: the next generation. Cell.

[9] Vander Heiden MG (2013). Exploiting tumor metabolism: challenges for clinical translation. J Clin Invest.

[10] DeBerardinis RJ, Thompson CB (2012). Cellular metabolism and disease: what do metabolic outliers teach us?. Cell.

[11] Ward PS, Thompson CB (2012). Metabolic reprogramming: a cancer hallmark even Warburg did not anticipate. Cancer Cell.

[12] Vander Heiden MG (2011). Targeting cancer metabolism: a therapeutic window opens. Nat Rev Drug Discov.

[13] Young VR, Ajami AM (2001). Glutamine: the emperor or his clothes?. J Nutr.

[14] Wullschleger S, Loewith R, Hall MN (2006). TOR signaling in growth and metabolism. Cell.

[15] Pingitore P, Pochini L, Scalise M, Galluccio M, Hedfalk K, Indiveri C (2013). Large scale production of the active human ASCT2 (SLC1A5) transporter in Pichia pastoris--functional and kinetic asymmetry revealed in proteoliposomes. Biochim Biophys Acta.

[16] di Salvo ML, Contestabile R, Paiardini A, Maras B (2013). Glycine consumption and mitochondrial serine hydroxymethyltransferase in cancer cells: the heme connection. Med Hypotheses.

[17] Jain M, Nilsson R, Sharma S, Madhusudhan N, Kitami T, Souza AL (2012). Metabolite profiling identifies a key role for glycine in rapid cancer cell proliferation. Science.

[18] Yuneva MO, Fan TW, Allen TD, Higashi RM, Ferraris DV, Tsukamoto T (2012). The metabolic profile of tumors depends on both the responsible genetic lesion and tissue type. Cell Metab.

[19] Diks SH, Peppelenbosch MP (2004). Single cell proteomics for personalised medicine. Trends Mol Med.

[20] Gygi SP, Rochon Y, Franza BR, Aebersold R (1999). Correlation between protein and mRNA abundance in yeast. Mol Cell Biol.

[21] Paweletz CP, Charboneau L, Bichsel VE, Simone NL, Chen T, Gillespie JW (2001). Reverse phase protein microarrays which capture disease progression show activation of pro-survival pathways at the cancer invasion front. Oncogene.

[22] Akbani R, Baker KF, Carragher N, Goldstein T, de Koning L, Korf U, et al. Realizing the promise of reverse phase protein arrays for clinical, translational and basic research: a workshop report. Mol Cell Proteomics. 2014. https://doi.org/10.1074/mcp.O113.034918 10.1074/mcp.O113.034918 PMC4083105 24777629

[23] Gonzalez-Angulo AM, Hennessy BT, Meric-Bernstam F, Sahin A, Liu W, Ju Z (2011). Functional proteomics can define prognosis and predict pathologic complete response in patients with breast cancer. Clin Proteomics.

[24] Boyd ZS, Wu QJ, O’Brien C, Spoerke J, Savage H, Fielder PJ (2008). Proteomic analysis of breast cancer molecular subtypes and biomarkers of response to targeted kinase inhibitors using reverse-phase protein microarrays. Mol Cancer Ther.

[25] Frederick MJ, VanMeter AJ, Gadhikar MA, Henderson YC, Yao H, Pickering CC (2011). Phosphoproteomic analysis of signaling pathways in head and neck squamous cell carcinoma patient samples. Am J Pathol.

[26] Sobin LH, Gospodarowicz MK, Wittekind C (2011). TNM classification of malignant tumours.

[27] Goldhirsch A, Wood WC, Coates AS, Gelber RD, Thürlimann B, Senn H-J (2011). Strategies for subtypes—dealing with the diversity of breast cancer: highlights of the St Gallen International Expert Consensus on the Primary Therapy of Early Breast Cancer 2011. Ann Oncol.

[28] von Minckwitz G, Untch M, Blohmer J-U, Costa SD, Eidtmann H, Fasching PA (2012). Definition and impact of pathologic complete response on prognosis after neoadjuvant chemotherapy in various intrinsic breast cancer subtypes. J Clin Oncol.

[29] Hudis CA, Barlow WE, Costantino JP, Gray RJ, Pritchard KI, Chapman J-AW (2007). Proposal for standardized definitions for efficacy end points in adjuvant breast cancer trials: the STEEP system. J Clin Oncol Off J Am Soc Clin Oncol.

[30] Henjes F, Bender C, von der Heyde S, Braun L, Mannsperger HA, Schmidt C (2012). Strong EGFR signaling in cell line models of ERBB2-amplified breast cancer attenuates response towards ERBB2-targeting drugs. Oncogenesis.

[31] Loebke C, Sueltmann H, Schmidt C, Henjes F, Wiemann S, Poustka A (2007). Infrared-based protein detection arrays for quantitative proteomics. Proteomics.

[32] Mannsperger HA, Gade S, Henjes F, Beissbarth T, Korf U (2010). RPPanalyzer: analysis of reverse-phase protein array data. Bioinforma Oxf Engl.

[33] Galili T (2015). dendextend: an R package for visualizing, adjusting and comparing trees of hierarchical clustering. Bioinforma Oxf Engl.

[34] Harrington DP, Fleming TR (1982). A class of rank test procedures for censored survival data. Biometrika.

[35] Modeling Survival Data: extending the Cox Model. Terry M. Therneau. Springer. http://www.springer.com/de/book/9780387987842. Accessed 9 Dec 2016.

[36] Benjamini Y, Hochberg Y (1995). Controlling the false discovery rate: a practical and powerful approach to multiple testing. J R Stat Soc Ser B Methodol.

[37] Szklarczyk D, Franceschini A, Wyder S, Forslund K, Heller D, Huerta-Cepas J (2015). STRING v10: protein-protein interaction networks, integrated over the tree of life. Nucleic Acids Res.

[38] R Core Team (2013). R: A Language and environment for statistical computing.

[39] Mertins P, Mani DR, Ruggles KV, Gillette MA, Clauser KR, Wang P (2016). Proteogenomics connects somatic mutations to signalling in breast cancer. Nature.

[40] Myhre S, Lingjærde O-C, Hennessy BT, Aure MR, Carey MS, Alsner J (2013). Influence of DNA copy number and mRNA levels on the expression of breast cancer related proteins. Mol Oncol.

[41] Wise DR, Thompson CB (2010). Glutamine addiction: a new therapeutic target in cancer. Trends Biochem Sci.

[42] Dolfi SC, Chan LL, Qiu J, Tedeschi PM, Bertino JR, Hirshfield KM (2013). The metabolic demands of cancer cells are coupled to their size and protein synthesis rates. Cancer Metab.

[43] Ren P, Yue M, Xiao D, Xiu R, Gan L, Liu H (2015). ATF4 and N-Myc coordinate glutamine metabolism in MYCN-amplified neuroblastoma cells through ASCT2 activation. J Pathol.

[44] Huang F, Zhao Y, Zhao J, Wu S, Jiang Y, Ma H (2014). Upregulated SLC1A5 promotes cell growth and survival in colorectal cancer. Int J Clin Exp Pathol.

[45] Wang Q, Hardie RA, Hoy AJ, van Geldermalsen M, Gao D, Fazli L (2015). Targeting ASCT2-mediated glutamine uptake blocks prostate cancer growth and tumour development. J Pathol.

[46] Liu Y, Yang L, An H, Chang Y, Zhang W, Zhu Y (2015). High expression of Solute Carrier Family 1, member 5 (SLC1A5) is associated with poor prognosis in clear-cell renal cell carcinoma. Sci Rep.

[47] Shimizu K, Kaira K, Tomizawa Y, Sunaga N, Kawashima O, Oriuchi N (2014). ASC amino-acid transporter 2 (ASCT2) as a novel prognostic marker in non-small cell lung cancer. Br J Cancer.

[48] Adeva MM, Souto G, Blanco N, Donapetry C (2012). Ammonium metabolism in humans. Metabolism.

[49] Marin M, Lavillette D, Kelly SM, Kabat D (2003). N-linked glycosylation and sequence changes in a critical negative control region of the ASCT1 and ASCT2 neutral amino acid transporters determine their retroviral receptor functions. J Virol.

[50] Nakaya M, Xiao Y, Zhou X, Chang J-H, Chang M, Cheng X (2014). Inflammatory T cell responses rely on amino acid transporter ASCT2 facilitation of glutamine uptake and mTORC1 kinase activation. Immunity.

[51] Fuchs BC, Bode BP (2005). Amino acid transporters ASCT2 and LAT1 in cancer: partners in crime?. Semin Cancer Biol.

[52] Wang Q, Beaumont KA, Otte NJ, Font J, Bailey CG, van Geldermalsen M (2014). Targeting glutamine transport to suppress melanoma cell growth. Int J Cancer.

[53] Willems L, Jacque N, Jacquel A, Neveux N, Maciel TT, Lambert M (2013). Inhibiting glutamine uptake represents an attractive new strategy for treating acute myeloid leukemia. Blood.

[54] Sitter B, Bathen TF, Singstad TE, Fjøsne HE, Lundgren S, Halgunset J (2010). Quantification of metabolites in patients with breast cancer with different clinical prognosis using HR MAS MR spectroscopy. NMR Biomed.

