# Supplementary figures and images for: Proteomic profiling of breast cancer metabolism identifies SHMT2 and ASCT2 as prognostic factors
Source: Breast Cancer Res. 2017 Oct 11;19:112. doi: 10.1186/s13058-017-0905-7 (PMC5637318; doi:10.1186/s13058-017-0905-7)

**OS: 2 Clusters**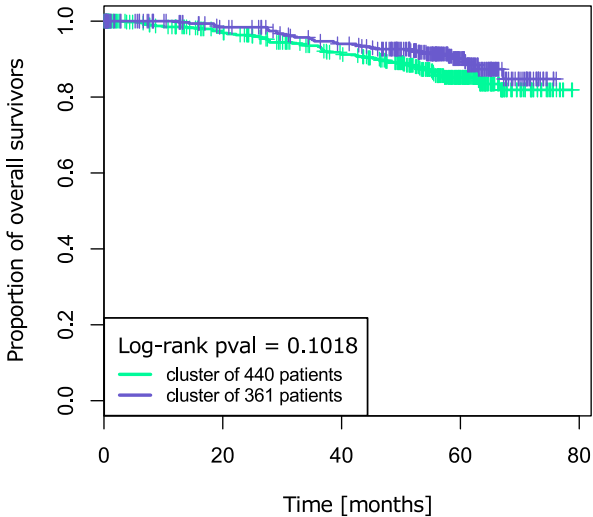**RFS: 2 Clusters**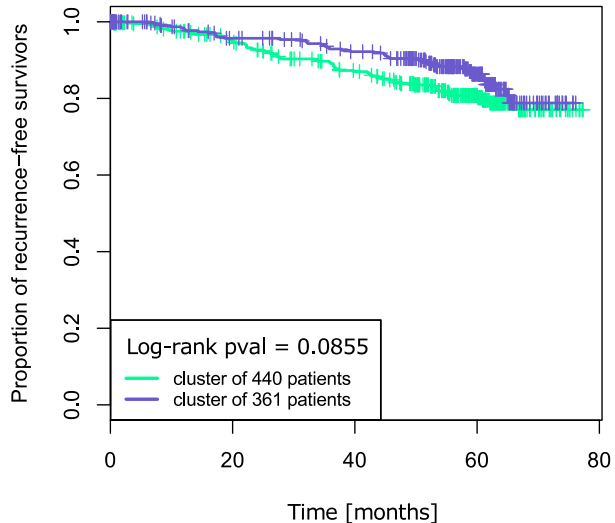

Supplement: Supplementary file 1 — Kaplan-Meier analysis of green and violet cluster. Kaplan-Meier curves show proportions of overall survivors (OS) and recurrence-free survivors (RFS) of two separate clusters. Statistical difference in outcome between Kaplan-Meier curves were compared by log-rank test. (PDF 93 kb) [file 13058_2017_905_MOESM1_ESM.pdf]
