# Supplementary material for: Proteomic profiling of breast cancer metabolism identifies SHMT2 and ASCT2 as prognostic factors
Source: Breast Cancer Res. 2017 Oct 11;19:112. doi: 10.1186/s13058-017-0905-7 (PMC5637318; doi:10.1186/s13058-017-0905-7)
Supplement: Supplementary file 2 — Protein network visualization. STRING illustrations are based on proteins represented in the “compact”’ cluster subgroup (A) and proteins represented in the “diffuse” cluster subgroup (B). STRING visualization was performed for each group individually and the evidence based network edges were set to an interaction score of 0.4. The given legend shows the type of interactions that were selected for the visualization. (PDF 2496 kb) [file 13058_2017_905_MOESM2_ESM.pdf]

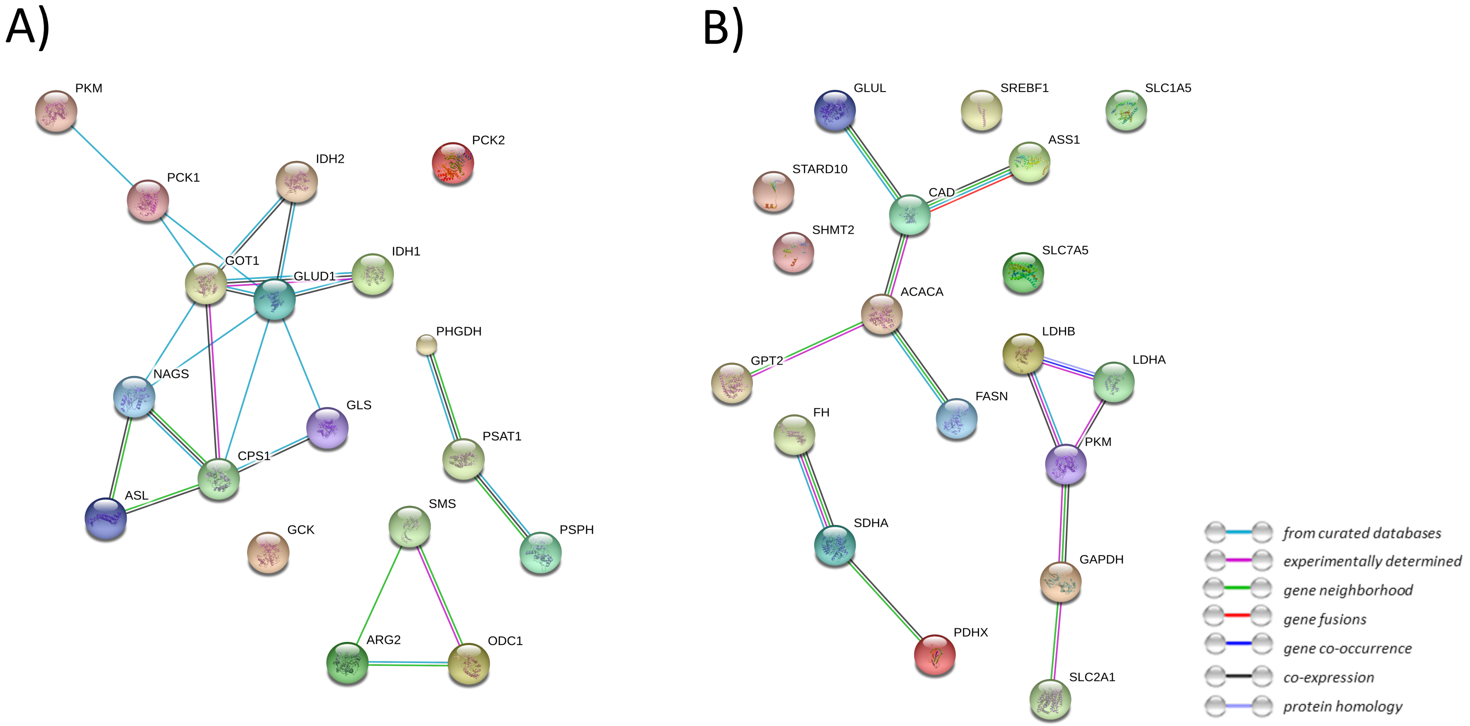
A)
PKM
PCK2
IDH2
PCK1
GOT1
GLUD1
IDH1
PHGDH
NAGS
GLS
CPS1
PSAT1
ASL
SMS
GCK
PSPH
ARG2
ODC1
B)
GLUL
SREBF1
SLC1A5
ASS1
STARD10
CAD
SHMT2
SLC7A5
ACACA
LDHB
GPT2
LDHA
FASN
FH
PKM
SDHA
GAPDH
PDHX
SLC2A1
from curated databases
experimentally determined
gene neighborhood
gene fusions
gene co-occurrence
co-expression
protein homology
